# Combined diffusion‐relaxometry microstructure imaging: Current status and future prospects

**DOI:** 10.1002/mrm.28963

**Published:** 2021-08-19

**Authors:** Paddy J. Slator, Marco Palombo, Karla L. Miller, Carl‐Fredrik Westin, Frederik Laun, Daeun Kim, Justin P. Haldar, Dan Benjamini, Gregory Lemberskiy, Joao P. de Almeida Martins, Jana Hutter

**Affiliations:** ^1^ Centre for Medical Image Computing Department of Computer Science University College London London UK; ^2^ Wellcome Centre for Integrative Neuroimaging, FMRIB, Nuffield Department of Clinical Neurosciences University of Oxford Oxford UK; ^3^ Department of Radiology Brigham and Women’s Hospital Harvard Medical School Boston MA USA; ^4^ Institute of Radiology University Hospital Erlangen Friedrich‐Alexander‐Universität Erlangen‐Nürnberg (FAU) Erlangen Germany; ^5^ Ming Hsieh Department of Electrical and Computer Engineering University of Southern California Los Angeles CA USA; ^6^ Signal and Image Processing Institute University of Southern California Los Angeles CA USA; ^7^ The Eunice Kennedy Shriver National Institute of Child Health and Human Development Bethesda MD USA; ^8^ The Center for Neuroscience and Regenerative Medicine Uniformed Service University of the Health Sciences Bethesda MD USA; ^9^ Center for Biomedical Imaging NYU School of Medicine New York NY USA; ^10^ Division of Physical Chemistry, Department of Chemistry Lund University Lund Sweden; ^11^ Department of Radiology and Nuclear Medicine St. Olav’s University Hospital Trondheim Norway; ^12^ Centre for Biomedical Engineering School of Biomedical Engineering and Imaging King’s College London London UK; ^13^ Centre for the Developing Brain School of Biomedical Engineering and Imaging King’s College London London UK

**Keywords:** diffusion, multidimensional MRI, quantitative MRI, relaxometry

## Abstract

Microstructure imaging seeks to noninvasively measure and map microscopic tissue features by pairing mathematical modeling with tailored MRI protocols. This article reviews an emerging paradigm that has the potential to provide a more detailed assessment of tissue microstructure—combined diffusion‐relaxometry imaging. Combined diffusion‐relaxometry acquisitions vary multiple MR contrast encodings—such as b‐value, gradient direction, inversion time, and echo time—in a multidimensional acquisition space. When paired with suitable analysis techniques, this enables quantification of correlations and coupling between multiple MR parameters—such as diffusivity, $T_{1}$, $T_{2}$, and $T_{2}^{*}$. This opens the possibility of disentangling multiple tissue compartments (within voxels) that are indistinguishable with single‐contrast scans, enabling a new generation of microstructural maps with improved biological sensitivity and specificity.

## 1 INTRODUCTION

This article reviews the current capabilities and future potential of an emerging paradigm in microstructure imaging: combined diffusion‐relaxometry. Diffusion MRI (dMRI) indirectly assesses tissue microstructure by measuring water diffusion.^1^ MR relaxometry, while also sensitive to small‐scale tissue structures,^2^ additionally offers information on the chemical composition of tissue^3^ through the estimation of transverse and longitudinal relaxation times. Conventionally, diffusion and relaxation properties are measured and analyzed *independently*. Combined diffusion‐relaxometry techniques measure and analyze diffusion and relaxation properties *jointly*, based on scans that vary both diffusion (eg, b‐value and gradient direction) and relaxation (eg, inversion time [TI], flip angle, repetition time [TR] and echo time [TE]) sensitizing sequence parameters in multiple combinations. This yields images where contrast reflects both diffusion properties (eg, diffusivity and anisotropy) and relaxation times (eg, $T_{1}$, $T_{2}$, $T_{2}^{*}$). The opening up of a multidimensional acquisition space enables exploration of the correlations between these complementary MR contrasts. Analyzing such multidimensional data with appropriate techniques can potentially reveal unique information on tissue microstructure. In particular, by identifying and disentangling the unique MR signatures of different components, we can precisely characterize multiple tissue environments within a single voxel.

In this article, we focus on imaging, but the concepts and approaches we discuss have a strong foundation in multidimensional NMR techniques developed in the context of porous media analysis. Such experiments typically sample diffusion and relaxation sequence parameters in a multidimensional acquisition space, and hence estimate multidimensional correlation spectra (ie, multivariate distributions of NMR properties). First used to estimate $T_{1}$‐$T_{2}$ distributions (eg, Ref. [4]) they were later extended to calculate $T_{2}$‐diffusivity distributions.^5^, ^6^, ^7^ The approach was further enabled by development of efficient techniques^8^, ^9^ for calculating multidimensional correlation spectra, allowing combined diffusion‐relaxometry NMR to be deployed in a wide variety of applications, including geology^10^, ^11^, ^12^ and food science.^13^, ^14^, ^15^ The uptake of these techniques shows that combining diffusion and relaxation information in multidimensional scans can provide heightened sensitivity to chemical composition and microstructural features (see references^16^, ^17^ for in depth reviews of multidimensional correlation NMR in porous media). The new insights into porous media demonstrated by these techniques motivated their translation to quantify microstructure in biological systems. Early work applied combined diffusion‐relaxometry to ex‐vivo biological systems, such as frog sciatic nerve,^18^ rat brain,^19^ yeast cells,^20^ and muscle^21^; alongside in vivo studies exploring the interrelationship between diffusion and relaxation properties in human brain.^22^

**FIGURE 1:**
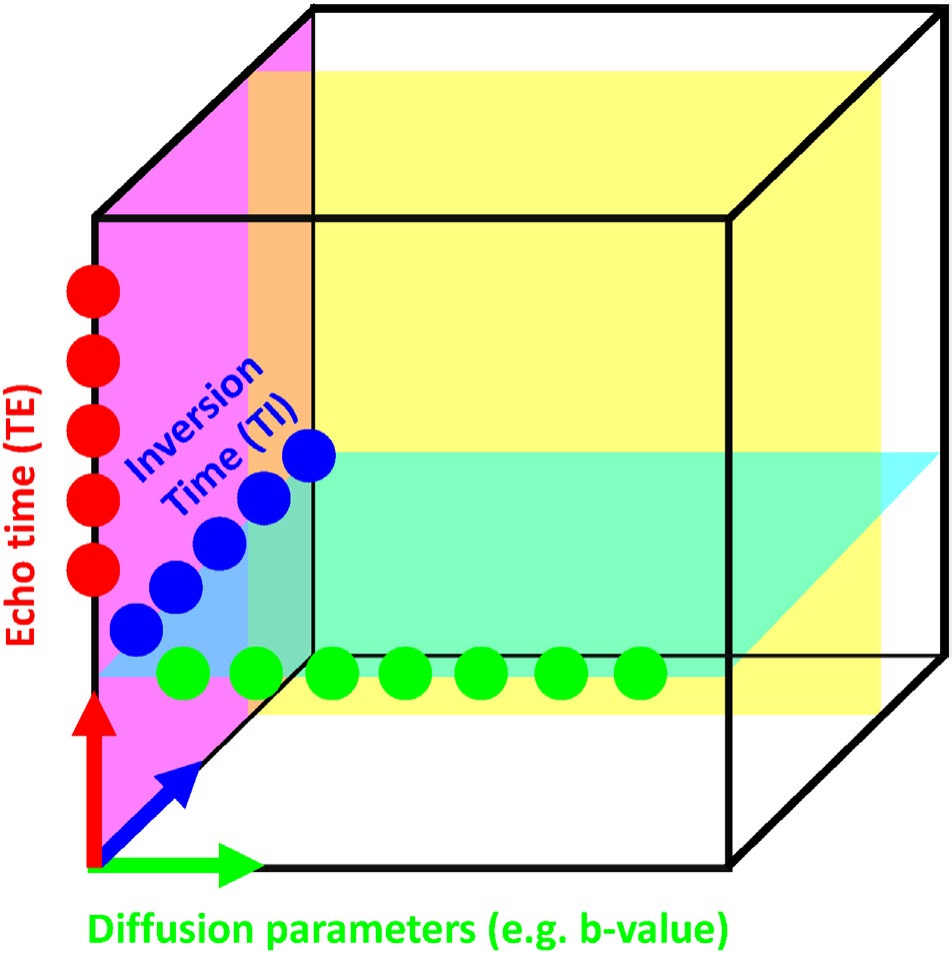
The parameter space relevant for combined diffusion‐relaxometry experiments. Diffusion parameters, for example, b‐value and gradient direction, are represented on the *x*‐axis. The echo times (TE) are on the y‐axis, and the inversion times (TI) on the *z*‐axis. The green dots represent a conventional diffusion acquisition at fixed TE with multiple diffusion preparations. The red dots illustrate a scan sampling several TEs without diffusion weighting to achieve $T_{2}$/$T_{2}^{*}$ maps. The blue dots illustrate a scan sampling multiple TIs without diffusion weighting to achieve $T_{1}$ maps. The transparent cyan, yellow and magenta planes depict the acquisition parameter space sampled in hypothethical $T_{1}$‐diffusion, $T_{2}$‐diffusion (equivalently $T_{2}^{*}$‐diffusion), and T1‐T2 experiments, respectively

**FIGURE 2:**
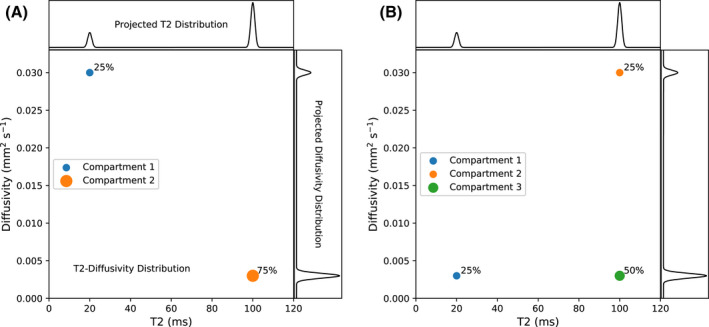
Separate diffusion and relaxation experiments can confound distinct tissue microenvironments. A,B, display simplified tissue structures comprising two and three distinct microenvironments, respectively. Each point denotes a distinct microenvironment with fixed $T_{2}$, diffusivity values, and a percentage volume fraction. The projected $T_{2}$ and diffusivity distributions are shown on the top and right‐hand sides. The 1D distributions are equivalent for both (A) and (B) despite the different tissue structures, showing that 1D measurements can confound distinct tissue microenvironments. Combined diffusion‐relaxometry can disentangle the contributions from the distinct microenvironments due to its ability to quantify correlations between multiple contrasts

There are two main strengths of combined diffusion‐relaxometry driving its expansion in the microstructure imaging field: (i) it accesses complementary measurements with the potential to separate and quantify multiple microstructural environments and (ii) it accounts for inherent biases in such measurements, which are present as relaxation properties of complex tissue environments in an MRI voxel influence the estimation of the corresponding diffusion properties. These biases stem from the intrinsic dependence of the diffusion MRI signal on sequence parameters, such as TE and TR, which hinders the separation and quantification of tissue compartments with distinct chemical and microstructural properties. For example, if $T_{1}$, $T_{2}$, and/or $T_{2}^{*}$ vary across tissue compartments, then volume fractions inferred from diffusion‐only scans will be additionally weighted by the corresponding relaxation times. This means that while it may be possible to satisfactorily quantify some microstructural environments by measuring multiple MR contrasts in a series of independent 1D acquisitions (ie, dots in Figure 1) other microstructural environments can only be comprehensively characterized by measuring multiple contrasts in combination, that is, by varying diffusion and relaxation encoding parameters in a 2D (ie, planes in Figure 1) or higher acquisition space. We highlight this with a $T_{2}$‐diffusion example in Figure 2. Two tissue structures are shown in (A) and (B), comprising two and three distinct microstructural compartments respectively. These tissue structures cannot be distinguished with single contrast experiments that estimate $T_{2}$ relaxation time and diffusivity separately. While there are numerous real‐world examples, in $T_{1}$,^23^ $T_{2}$ ^24^, $T_{2}^{*}$ ^25^ and diffusion^1^ domains, where tissue microenvironments can be distinguished with single‐contrast experiments, combined multicontrast experiments may be required to fully assess the tissue of interest. An example is the proposed separation of intra‐ and extra‐axonal compartments in the brain at clinically accessible diffusion weightings ($b\leq 3\text{ms}/\mu m^{2}$), which is facilitated by combining both $T_{2}$ and diffusivity measures ^26^; using only relaxation or diffusion data separately leads to possible failure of the model to represent reality and/or to very high uncertainty on the intra‐axonal vs extra‐axonal estimates.

This review provides an introduction to combined diffusion‐relaxometry imaging with focus on three overarching themes: acquisition, analysis, and applications. Suitable acquisition techniques are crucial for diffusion‐relaxometry, particularly to address the severe increase in scan time that moving from 1D to 2D (and higher dimensions) necessitates. The acquisition section reviews the additional contrast encoding parameters that can be combined with diffusion to make a diffusion‐relaxometry experiment, including TE in spin echo and gradient echo sequences, and TI in inversion recovery sequences. We also review additional diffusion‐weighting parameters beyond b‐values and gradient directions that can be combined with relaxometry, such as diffusion time, b‐tensor shape, mixing time, and B0 field strength. Given these higher dimensional datasets, new analysis techniques that account for correlations between relaxation and diffusion properties can offer exciting new perspectives on tissue microstructure. We discuss the range of modeling approaches in the analysis section, including continuum modeling methods that make minimal assumptions about tissue structure to calculate multidimensional correlation spectra, other signal representation approaches based on the cumulant expansion, and microstructure modeling approaches where a fixed number of water pools are assumed a priori. In line with the ultimate goal of combined diffusion‐relaxometry techniques that make biologically and clinically meaningful observations, in the final section we review application areas where diffusion‐relaxometry techniques have contributed novel insights.

## 2 ACQUISITION

We first review techniques suitable for acquiring combined diffusion‐relaxometry data. The essential property of such techniques is that they sample a multidimensional parameter space including diffusion (eg, b‐value) and relaxation (eg, TE, TI) encoding parameters. This section proceeds as follows. In “Background and Motivation,” we first explain a significant limitation of dMRI—the confounds that diffusion preparations introduce into MRI acquisition sequences and the resulting intertwined relationship between dMRI and relaxation, then introduce some basic combined diffusion‐relaxometry acquisition strategies. We next introduce and discuss advanced acquisition techniques that seek to acquire more informative diffusion‐relaxometry data with higher efficiency in “Advanced combined diffusion‐relaxometry acquisition.” We finish by introducing some advanced diffusion MRI and MR relaxometry techniques that are particularly relevant for combined diffusion‐relaxometry in “Combined diffusion‐relaxometry and complimentary advanced acquisition techniques.”

### 2.1 Background and motivation

#### 2.1.1 Intrinsic relaxation in diffusion MRI experiments

**FIGURE 3:**
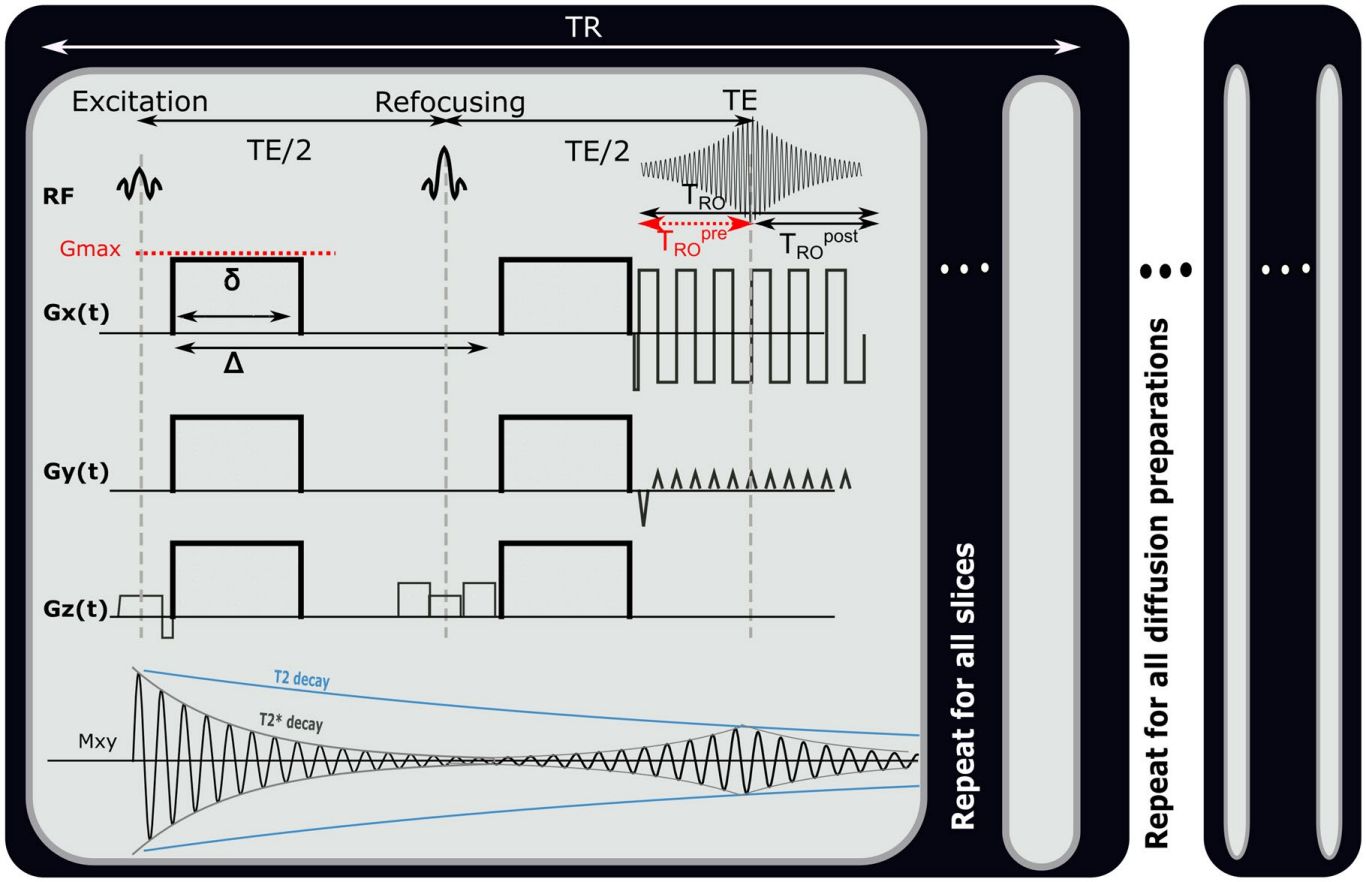
Standard pulsed gradient spin echo (PGSE) ssEPI dMRI acquisition. From top down the pulse diagram shows: the excitation and refocusing RF pulses, a standard diffusion preparation in all three directions alongside the EPI read‐out train, and the $T_{2}$ and $T_{2}^{*}$ decay occurring during the spin echo acquisition. A whole dMRI scan comprises multiple repeats of these PGSE ssEPI blocks, one for each slice and each diffusion preparation used. The repetition time TR is defined as the time required to sample each slice in one stack. $T_{\mathrm{RO}}$ denotes the readout time, $G_{\max}$ the available gradient strength, δ the length of the gradient lobe, and Δ the separation of the gradient pulses

The standard technique for dMRI is single‐shot echo planar imaging (ssEPI). In ssEPI the diffusion preparation is followed by an EPI read‐out train as shown in Figure 3. In this illustrated example, the diffusion preparation, described by the gradient waveform, is a standard pulsed gradient experiment (ie, pulsed gradient spin echo [PGSE]) with two gradient lobes of equal length δ, equal polarity, and equal gradient strength $G_{\max}$. One lobe lies between the excitation and refocusing pulse, with the other between the refocusing pulse and the start of the EPI read‐out. The spacing between the gradient lobes (Δ) determines the diffusion time. In this classic setup, the b‐value is determined by $b=\gamma^{2}G^{2}\delta^{2}\left(\Delta ‐\delta /3\right)$, a special case of the more generic formulation

(1) $$\begin{array}{c} b=\int_{0}^{t}q{\left(t^{\prime }\right)}^{2}dt \end{array}$$

where

(2) $$\begin{array}{c} q\left(t\right)=\gamma \int_{0}^{t}G\left(t^{\prime }\right)dt. \end{array}$$

$G\left(t\right)={\left[G_{x}\left(t\right)\;G_{y}\left(t\right)\;G_{z}\left(t\right)\right]}^{T}$ describes the gradient waveform, and γ denotes the field‐strength‐dependent gyromagnetic ratio over 2π. Typically the echo time (TE) is minimized, which achieves the highest possible SNR. Therefore, the key limitations are the length of EPI read‐out train before the TE ($T_{\mathrm{RO}}^{\mathrm{pre}}$ in Figure 3), and the available gradient strength $G_{\max}$. Figure 3 also illustrates that the diffusion acquisition does not happen in isolation, but in parallel to the $T_{2}$/$T_{2}^{*}$ decay. It follows that the measured ssEPI signal is influenced both by $T_{2}$ decay and by the effect of diffusion gradients and can be written, assuming monoexponential relaxation and diffusion decays

(3) $$\begin{array}{c} S\left(b,TE\right)=M_{0}e^{‐TE/T_{2}}e^{‐bD} \end{array}$$

where *D* is the apparent diffusion coefficient and $T_{2}$ is the $T_{2}$ relaxation time. It follows that the choice of the highest b‐value in a dMRI acquisition, and hence the minimum TE possible due to the restrictions outlined above (assuming fixed TE across all volumes), has a direct effect on the signal attenuation *for all b‐values* in the acquisition. Therefore, these choices influence quantitative diffusion‐related metrics derived from dMRI experiments. Two artifacts arising from the influence of the transverse relaxation are $T_{2}$ shine‐through and $T_{2}$ blackout, where variations in the $T_{2}$ time (eg, in hematomas or myelin water) influence the dMRI signal intensity.^27^

#### 2.1.2 Basic combined diffusion‐relaxometry acquisition

The inherent relationship between diffusion and relaxation acquisition sequences, alongside the opportunity to probe tissue microstruture in more detail, motivates MRI acquisition sequences that probe diffusion and relaxation properties and their correlations. We will now introduce and review such acquisition sequences. For clarity, here and throughout we avoid using the terms “multicontrast” and “multimodal”, which can refer to any scan (or series of individual scans) that measures multiple MR contrasts. Instead we use “combined diffusion‐relaxometry,” to mean an experiment where diffusion and relaxation encoding parameters are varied in a 2D (or higher) acquisition space (eg, a diffusion‐prepared sequence repeated at multiple TEs), and “simultaneous combined diffusion‐relaxometry” for the special case where multiple diffusion and relaxation encoding parameters are varied within a single repetition time (TR) (eg, diffusion‐prepared sequence where multiple TEs are measured in a single TR).

##### Consecutive acquisitions

**FIGURE 4:**
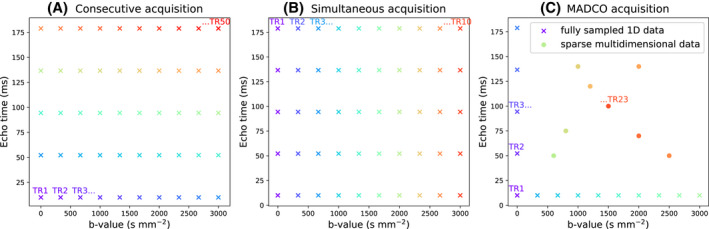
Example $T_{2}$‐diffusion acquisitions with three existing techniques. Each color represents a distinct excitation of length TR. A, The consecutive approach: repeat diffusion‐prepared scans with different TEs (illustrative acquisition time: 50 TRs). B, Simultaneous approach: applying techniques allowing multiple samples in the parameter space within the same excitation of length TR (illustrative acquisition time: 10 TRs). C, MADCO approach^58^: Fully sampled 1D data are augmented with sparsely sampled multidimensional data (illustrative acquisition time: 23 TRs). While we only show 2D $T_{2}$‐diffusion (or $T_{2}^{*}$‐diffusion) experiments for convenience, the same principles apply when extending these techniques to 3D and higher

**FIGURE 5:**
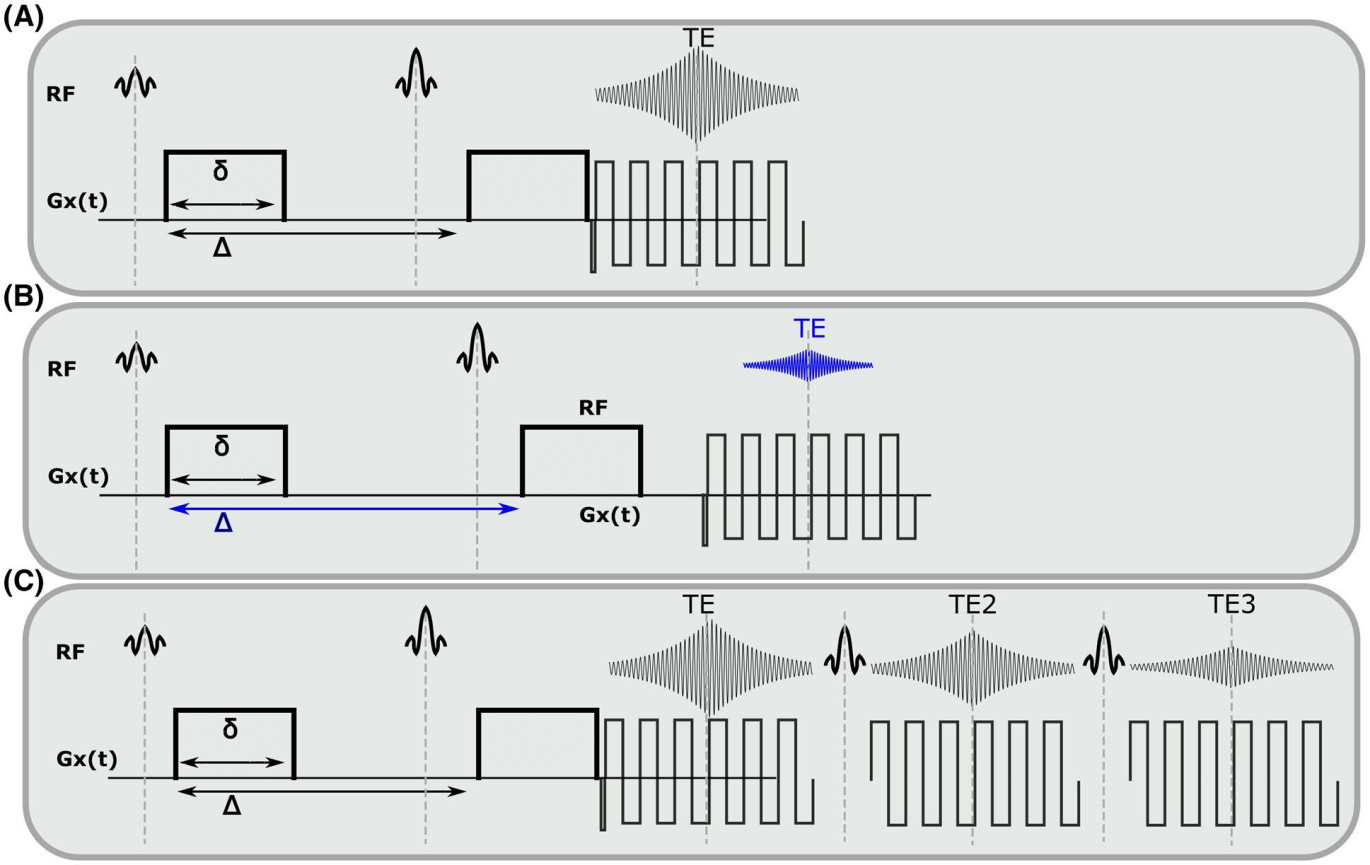
Illustration of separate vs integrated acquisition strategies using the example of multiple diffusion‐encoded scans with varying TE, that is, $T_{2}$‐diffusion. A,B, show separate acquisitions at different TEs, together with the included dead time and change in diffusion preparation. Acquiring (A) and (B) to sample the 2D space is a basic ‘consecutive’ acquisition. Integrated multi‐echo acquisitions are shown in (C) for spin echoes, hence acquiring combined diffusion‐relaxometry data ‘simultaneously’

The most common way to acquire combined diffusion‐relaxometry data is by repeating a diffusion‐weighted scan with varying relaxation‐encoding parameters. For example, since $T_{2}$ relaxation time can be estimated by acquiring multiple spin echoes with different TEs,^28^ typical $T_{2}$‐diffusion experiments comprise acquiring multiple diffusion prepared spin echoes with varying TEs (eg, Figure 4A). With this approach, each sample in the high‐dimensional acquisition space (eg, Figures 1 and 4) is obtained with its own acquisition block of length TR. This is inefficient, as the time per sample is limited by the time required to achieve the specific diffusion preparation. This can lead to large idle times compared to typical dMRI—where TE is minimized subject to the constraints of the required b‐value and imaging gradients (eg, Figure 5A)—as data are acquired at a range of TEs with some longer than the minimal TE (eg, Figure 5B). Consequently, typical consecutive combined diffusion‐relaxometry $T_{2}$‐diffusion acquisitions, such as in Figure 4A, have taken 1 hour to scan the whole brain with around 500 volumes at 2.5 mm isotropic resolution.^26^, ^29^ As well as being slow and inefficient, this approach adds to the risk of inconsistencies and bias through motion and modifying the diffusion time.

**FIGURE 6:**
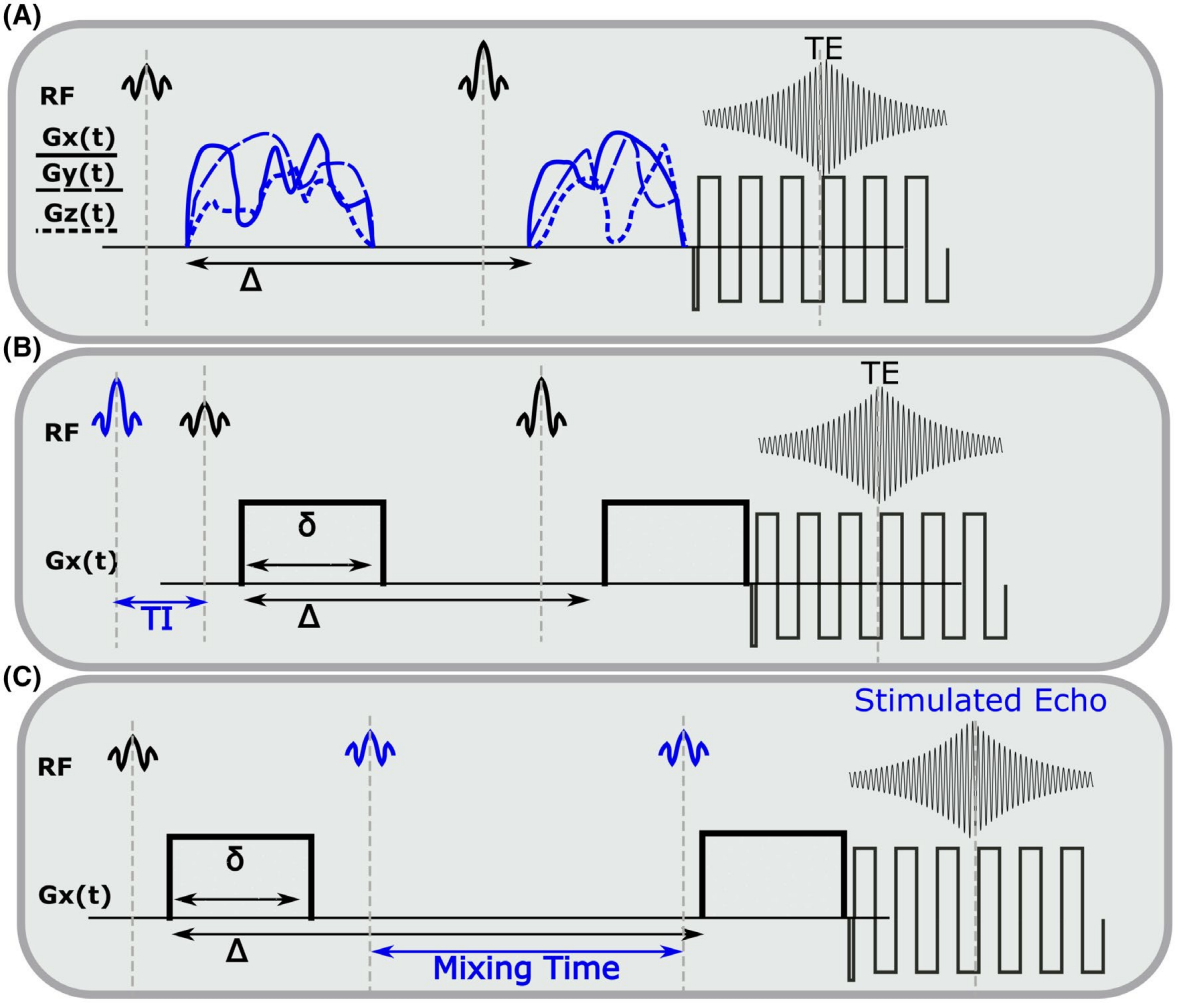
Illustration of several alternatives to the conventional PGSE acquisition. A, Replacing the bipolar gradient pair by free waveforms used for q‐space sampling. B, Addition of a global inversion pulse to achieve a set inversion time TI. C, STEAM acquisition including and depicting the mixing time, introducing $T_{1}$ sensitivity and changing the diffusion time

The effect for $T_{1}$‐diffusion experiments is even more drastic. A basic $T_{1}$‐diffusion acquisition comprises spin echo acquisitions preceded with a global $180^{\circ }$ inversion pulse, as in a typical inversion recovery sequence,^30^ with the time between the global inversion pulse and the first spin echo pulse (the inversion time, TI) varied to yield $T_{1}$ sensitivity. This introduces significant delays compared to typical dMRI sequences, as the time between inversion and excitation, which sets the achieved TI (see Figure 6B), is typically in the range of 0‐3000 ms. Moreover, the inversion recovery sequence is typically performed slice‐by‐slice, with the global inversion pulse followed by reading out a single slice. An early in vivo $T_{1}$‐diffusion combined diffusion‐relaxometry experiment gave whole brain $T_{1}$‐diffusion coverage with 2 mm isotropic voxels in 1 hour.^31^

This intrinsic inefficiency in sequences, together with the huge number of possible data points—two previous 2D diffusion‐relaxometry studies using uniform sampling utilized 1024^32^ and 225 000^33^ data points—hampers multidimensional scanning, and in the past prevented translation of such techniques from NMR to imaging. It is therefore clear that methods for faster and more efficient combined diffusion‐relaxometry acquisition protocols are of paramount importance. Two commonly employed strategies for working toward this goal are:

1. modifying the pulse sequence to acquire data points more efficiently (ie, efficient sampling schemes)
2. selecting acquisition parameters that maximize the information content for subsequent analysis and interpretation (ie, optimized sampling schemes)

### 2.2 Advanced combined diffusion‐relaxometry acquisition

#### 2.2.1 Efficient sampling schemes

There are multiple techniques that apply the aforementioned first strategy, often inspired by efficient simultaneous diffusion and relaxation acquisitions pioneered in the NMR domain.^34^, ^35^, ^36^ These bespoke MR sequences typically improve acquisition efficiency by combining multiple contrast preparations and multiple read‐outs within a single TR. In particular, for transverse relaxometry, time can be saved by acquiring multiple echoes (either spin or gradient) after the initial diffusion preparation and spin echo readout (eg, Figure 4B). This is further illustrated for the multiple spin echo case, which captures $T_{2}$ information, in Figure 5C. Similarly, additional gradient echoes would capture $T_{2}^{*}$ information, akin to a typical multi‐echo gradient echo scan for measuring $T_{2}^{*}$ relaxation.^37^ In both spin and gradient echo cases, this approach means that a single diffusion preparation is shared across multiple echoes, rather than being repeated for each echo, with the benefits of a more efficient scan and a consistent diffusion preparation across echoes. This results in a more complete sampling of the TE‐diffusion preparation space within a fixed scan time. The exact speed up depends on the number of TEs chosen, and the required extension of the shot length and the duty cycle requirements set by the heating of the gradients. For large b‐values, the TR might be extended due to this requirement of cooling periods, potentially offsetting some time gains when acquiring multiple echoes.

For $T_{1}$ relaxometry experiments where a global inversion pulse is inserted before the first excitation (ie, Figure 6B), again there are NMR‐inspired techniques that more efficiently sample multiple TIs over the imaging volume. Typical inversion recovery experiments acquire multiple spatial slices sequentially, meaning that each slice has a different effective TI value, allowing the measurement of many points on the inversion recovery curve, albeit at different spatial locations, within a single TR. The addition of slice‐shuffling as originally proposed in 1990 and recently done in a number of studies,^38^, ^39^, ^40^ where the acquisition order of spatial slices is changed in subsequent TRs, allows the efficient sampling of each TI at each spatial slice location. Efficient slice shuffling has recently been utilized to yield whole brain T1‐diffusion coverage in 13 minutes for 2.6 mm isotropic voxels.^40^ An alternative approach avoids inversion recovery altogether by only varying TR to yield T1 sensitivity.^41^

Further acquisition flexibility comes from applying the slice shuffling principle to the diffusion preparation, as demonstrated recently by Hutter et al^42^ who, instead of acquiring all spatial slices with the same diffusion preparation in a single TR, changed the diffusion preparation on a slice level. The ZEBRA (Z‐location shuffling, multiple echoes and B‐interleaving for relaxometry‐diffusion acquisitions) technique combines three strategies^42^, ^43^—multiple subsequent gradient echoes are combined with slice‐shuffling and interleaved slice‐level diffusion encoding. This allows the acquisition parameter spaces required for $T_{1}$, $T_{2}^{*}$, and diffusion contrast to be efficiently sampled in a significantly reduced acquisition time—a ZEBRA whole‐brain $T_{1}$‐$T_{2}^{*}$‐diffusion scan with 1344 volumes at 2.5 mm isotropic resolution was demonstrated in a total scan time of 52 minutes.^44^ However, there are remaining challenges and constraints for such pulse sequences: acquisition times are still too long for clinical translation, the number of subsequent echoes that can be acquired is constrained by the length of the EPI read‐out train and crusher gradients, and the acquired data cannot be easily split into shells—as each gradient direction will have different TIs due to slice shuffling—preventing the use of standard analysis techniques.

Stimulated echo (STEAM) techniques, albeit commonly employed to alter the diffusion time, also have an inherent $T_{1}$ sensitivity,^45^ although 50% of the signal is lost compared to a spin echo in the conventional setup (see Figure 6C for a representative STEAM sequence). However, a recent example exploiting stimulated echo pathways, MESMERISED (Multiplexed Echo Shifted Multiband Excited and Recalled Imaging of STEAM Encoded Diffusion), uses echo‐shifting to remove the dead time in STEAM acquisitions and hence allows more efficient $T_{1}$‐diffusion acquisition.^46^

Similarly, there are multiple techniques proposed to acquire transverse relaxometry data more efficiently. These include echo planar time‐resolved imaging (EPTI),^47^ which uses novel sampling strategies to exploit correlations in k‐space and time to enable faster $T_{2}$ and $T_{2}^{*}$ relaxometry. This approach was recently demonstrated for diffusion‐relaxometry with PEPTIDE (PROPELLER EPTI with dynamic encoding).^48^ Also related are high strength gradient systems, as they can enable lower TEs for the same diffusion weighting ^49^ although it does remain the case that lower minimum TEs are possible in 1D relaxometry experiments than combined diffusion‐relaxometry acquisitions. This may inhibit the ability of diffusion‐relaxometry to measure structures with low $T_{2}$/$T_{2}^{*}$ relaxation times, such as myelin water.^2^ magnetic resonance fingerprinting (MRF) is another relevant technique. This allows simultaneous quantification of multiple tissue properties by combining a highly accelerated acquisition that varies all relevant parameters, with dictionary matching to prior computed signal curves. The simulation of the MRF dictionary^50^ with additional parameters constitutes a large computational effort, limiting diffusion MRF so far largely to variation of the b‐value to allow calculation of the ADC value, for example, Ref. [51]. However, recent efforts have started to overcome this limitation by supporting this step with deep learning techniques, resulting in combined diffusion‐relaxometry MRF^52^ including variation of b‐value and gradient direction. Another proposed alternative is MR multitasking, a continuous acquisition that uses using real‐time low‐rank modeling to account for motion, relaxation and other dynamics and hence efficiently quantify MR properties,^53^ and was recently demonstrated for simultaneous $T_{1}$, $T_{2}$, and ADC mapping.^54^

#### 2.2.2 Optimized sampling schemes

In addition to sequence efficiency, the choice of acquisition parameters is of key importance. Sampling the acquisition parameter space (ie, Figures 1 and 4) with a uniform grid is the obvious place to start. However, similar to nonuniform k‐space sampling strategies, this is not required for analysis and not necessarily the most efficient approach. For example, the Cramer‐Rao lower bound (CRLB) is an estimation theoretic tool that can be used to quantify the efficiency of different encoding schemes,^55^ and CRLB‐based analysis suggests that there can be many sampling locations which provide little additional information within a uniform diffusion‐relaxometry sampling grid.^56^ Samples that do not provide much information can be skipped to enable high‐quality data from a very small number of samples. This approach has enabled estimation of multidimensional spectra from as few as 12 samples for 2D diffusion‐relaxation,^56^ and fitting of a two compartment $T_{2}$‐diffusion model from a 15 minutes whole brain scan at 2.5 mm isotropic resolution.^57^

The MADCO (marginal distributions constrained optimization) framework is another example of the reduced‐sampling approach. It exploits the fact that lower dimensional distributions, or *spectra*, of MR parameters estimated from the data are essentially projections of the corresponding multidimensional spectra. MADCO utilizes a hierarchical encoding scheme: first, fully sampled 1D data is acquired for all sampling dimensions; then 2D (or higher) datapoints are very sparsely sampled (see Figure 4C). This, when combined with analysis techniques detailed in later sections, supports estimation of multidimensional spectra using many less data points that would typically be required—MADCO was shown to achieve an acceleration factor of up to 50.^58^, ^59^ MADCO also has the significant advantage that acquisition times do not dramatically increase for higher dimensions, as demonstrated for the 3D case.^60^

Further acceleration techniques that reduce the number of required samples, all unified by the assumption that the data is sparse, have been proposed. While strategies like MADCO leverage the marginalized axes to constrain where peaks can appear, compressed sensing approaches—traditionally applied in k‐space—impose a presumed basis set in which the data is sparse. These methods have been used to recover 2D spectra using significantly less data in NMR^61^ and MRI^62^ contexts. Similarly, a framework based on PCA‐like optimization has been proposed^63^ and aims to retrieve the sparse basis from the data. Finally, recent frameworks using machine learning techniques such as SARDU‐net^64^ have shown excellent ability to retrieve information based on fewer samples.^44^

### 2.3 Combined diffusion‐relaxometry and complementary advanced acquisition techniques

The standard PGSE sequence discussed above can be modified in various ways to alter the sensitivity to specific microstructural features. In the following sections, we introduce some common modifications, and discuss their influence on relaxation properties. Since the temporal profile of diffusion gradients is directly linked to the information encoded into the signal, different gradient waveforms can expose (or suppress) different aspects of diffusive motion, such as restriction,^65^ flow,^66^, ^67^ anisotropy^68^ and exchange.^69^

#### 2.3.1 Tensor‐valued diffusion encoding schemes

Arbitrary diffusion‐weighting gradient waveforms (eg, Figure 6A) can be described in terms of a symmetric second‐order b‐tensor defined as^70^

(4) $$\begin{array}{c} B=\int_{0}^{t}q\left(t\right)q^{T}\left(t\right)dt, \end{array}$$

where *q* is defined in Equation (2).

The tensor‐valued diffusion encoding framework describes protocols where diffusion encoding is executed in more than one direction per signal preparation to enable the measurement of b‐tensors with arbitrary traces (ie, b‐values), shapes and orientations.^70^ In contrast, diffusion encoding schemes based on the Stejskal–Tanner design can only modulate the trace and orientation of the b‐tensor. Consequently, the benefit of tensor‐valued encoding is that it unlocks the “shape of the b‐tensor,” a new encoding dimension that can be modulated to control the sensitivity of the detected signal to microscopic diffusion anisotropy, as reviewed in detail in Ref. [71].

The parallels between novel diffusion gradient waveforms and combined diffusion‐relaxometry are clear—both are acquisition techniques that can precisely quantify and disentangle distinct tissue microenvironments and, excitingly, both approaches are complementary. It follows that modified gradient waveforms are necessary in order to maximize the utility of combined diffusion‐relaxometry acquisitions (and vice versa). For example, a sequence combining tensor‐valued diffusion encoding with multiple TR weighting was introduced as a means to establish correlations between $T_{1}$ and diffusion properties and thus characterize complex fiber orientation layouts.^41^ However, the combined relaxation and b‐tensor protocols developed thus far have utilized a rather straightforward design, particularly from the relaxation encoding perspective, with suboptimal echo and recovery time samplings. In this regard, there is potential for improvement through combining arbitrary b‐tensor shape with acquisition strategies that encode relaxation information more efficiently. We also note that, particularly when combined with relaxometry, the additional degrees of freedom describing an advanced diffusion gradient waveform put further emphasis on efficiently selecting the acquisition parameters.

#### 2.3.2 Varying diffusion time

As well as modifying diffusion gradient shape, we can also modify the time scale at which diffusion is observed. In the conventional PGSE approach, Δ, the spacing between diffusion encoding pulsed gradients, can be varied for this purpose. Importantly, Δ is closely linked to a change in TE as described above, due to the intertwining between the structure used for the spin echo and the placement of the diffusion gradients after the excitation and after the refocusing pulse.

There are a number of advanced acquisition techniques which probe the diffusion time. Higher gradients with faster slew rates can be used to allow MRI systems to access short Δ and may be critical for measurement of white matter features, such as the axonal diameter.^72^ Alternative sequences, such as the oscillating gradient spin echo (OGSE) are associated with significantly shorter effective diffusion times.^73^ For structures with larger radii (prostate lumen,^74^ muscle tissue^75^), PGSE cannot offer long enough diffusion times, due to signal loss from $T_{2}$‐weighting.

The aforementioned STEAM diffusion preparations (Figure 6C) can maintain high SNR for diffusion times above 50 ms. Although STEAM conflates $T_{1}$ and diffusion time dependencies, this may be overcome with a twice‐refocused STEAM preparation.^76^ In combination with the appropriate models (as discussed in later sections) this approach can investigate the correlations between the time‐dependence of diffusivity and relaxation times.

Modifying the diffusion time yields particular sensitivity to porous structures, such as cells.^77^ Such structures also affect relaxation properties, for example, differences in magnetic susceptibility between the pores and surrounding material cause spatial variations in the $B_{0}$ field.^78^ Song et al demonstrated that this can be exploited using sequences that vary the TE and diffusion time to measure the decay due to diffusion in the internal field (DDIF), and hence determine the characteristic length‐scales of a porous structure.^79^ Alvarez et al developed a related sequence sensitive to diffusion and relaxation properties to measure internal gradient distribution tensors, which probe local magnetic susceptibility properties.^80^

These experiments show that incorporating diffusion time dependence into combined diffusion‐relaxometry models is an exciting prospect for quantifying microscopic structures. Alongside magnetic susceptibility, future acquisition methods could also probe $T_{1}$ and $T_{2}$ relaxation. These methods can potentially detect compartments with small (∼1 μm) and impermeable features, which are not experimentally accesible with diffusion‐only sequences due to the limited diffusion times available on clinical MR scanners (20‐50 ms).

#### 2.3.3 $B_{0}$ field strength

Higher B0 field strength offers significant advantages for combined diffusion‐relaxometry, including increased SNR, contrast, and spatial and temporal resolution.^81^ However, relaxation times vary strongly with B0, and the specific relationship often depends on the tissue type, such as with $T_{1}$ in the brain.^82^ Since they are affected by relaxation, diffusion metrics may also depend on $B_{0}$,^83^ particularly at ultra‐high field (ie, 7T and above).^84^, ^85^ In some cases, it may be important to account for these dependencies in the clinic, such as when setting cut‐off values between healthy and diseased tissue. There are also likely opportunities to exploit this dependency—in some applications distinguishing between tissue types may be easier at a certain $B_{0}$. Ultra‐high‐field MRI seems most promising for revealing and exploiting $B_{0}$ dependencies of diffusion metrics. In particular, the reported increase of micro‐FA at ultra‐high‐field deserves further investigation.^85^, ^86^ Another promising avenue is the B0 dependency of the intravoxel incoherent motion (IVIM) signal curve at ultra‐high‐field,^87^, ^88^ where the weight of the arterial pool should further increase, combining this with additional relaxometry information can potentially make “arterial‐pool‐weighted” IVIM feasible.^89^

#### 2.3.4 Exchange

During acquisitions, some spins will inevitably move between microenvironments that have different MR properties. Traditional diffusion‐relaxometry acquisition techniques do not explicitly account for exchange or provide a means to modulate its effects, but exchange will affect the signal and hence derived diffusion and relaxation parameters. A class of related and relevant techniques can detect exchange between compartments using sequences incorporating variable mixing times. These include diffusion exchange spectroscopy (DEXSY)^69^ and filter exchange imaging (FEXI)^90^, ^91^ for diffusion, and relaxation exchange spectroscopy (REXSY) for $T_{2}$ ^92^ and $T_{1}$ ^93^ relaxation. Such techniques can only measure the apparent exchange rate, as differences in relaxation times (for diffusion exchange) or diffusivities (for relaxation exchange) between compartments will affect the measured exchange rate.^94^ Techniques that acquire combined diffusion‐relaxometry data with variable mixing times have the potential to account for these differences, and hence measure exchange rates more closely related to underlying values. However, naively merging these techniques would lead to prohibitively long acquisition times, so novel pulse sequences, such as methods that rapidly measure exchange,^95^ are desirable.

#### 2.3.5 Final thoughts

Further improvements will come from combining specific multidimensional data acquisition techniques with important parallel developments in MRI acceleration. These include multiband imaging^96^, ^97^ and compressed sensing approaches^62^, ^98^ among others. In summary, the efficient acquisition of combined diffusion‐relaxometry data is an exciting and ongoing field which will continue to be driven and influenced by a multitude of developments in both MRI and NMR. Such accelerated acquisition techniques are crucial to provide data for the appropriate analysis method while avoiding prohibitively long acquisition times.

## 3 ANALYSIS

### 3.1 Background and motivation

The basic premise of a combined diffusion‐relaxometry MRI experiment is that we are interested in understanding and quantifying the different microenvironments that coexist within an imaging voxel, under the assumption that these different microenvironments have distinctive diffusion‐relaxation characteristics that will allow them to be clearly discriminated from one another. The main goal of data analysis is therefore to identify and quantify different microenvironments. However, practical limits on the spatial resolution of MRI often mean that the signal from a single voxel will represent a partial‐volume mixture of multiple distinct microenvironments.

In the absence of exchange, the measured data can be modeled as the linear superposition of the signals that would be observed from each individual component, and data analysis generally necessitates some form of multicomponent modeling so that the contributions of different microenvironments can be disentangled. One of the main advantages of combined diffusion‐relaxometry is that the resulting multicomponent data analysis can be shown to be easier, both empirically^4^, ^99^, ^100^ and theoretically,^55^ than multicomponent diffusion analysis (without relaxation) or multicomponent relaxometry (without diffusion). Multiexponential fitting for a single parameter such as diffusivity or relaxation time is highly ill‐posed. However, joint exponential fitting for multiple parameters in 2D (or higher) is better conditioned than the corresponding 1D fits.

Separating a mixture of superposed signal components is a classical inverse problem that is not only unique to diffusion and relaxation MRI, but also appears commonly in other applications like remote sensing, spectroscopy of all kinds (including NMR), functional imaging (eg, fMRI, MEG, EEG, and PET), and dynamic contrast‐enhanced imaging (eg, MRI and PET). Due to its ubiquity, this kind of problem has been widely investigated over many decades, and a wide variety of analysis tools have been developed. Due to space limitations, the description below focuses on some of the most common unmixing approaches that are applicable to combined diffusion‐relaxometry, and is not intended to be comprehensive.

For the sake of concreteness and without loss of generality, our description will assume a simple MRI experiment that combines 1D diffusion encoding with $T_{2}$ relaxometry encoding, where each data sample is associated with one diffusion‐encoding parameter b, one relaxation‐encoding parameter TE, and one spatial position (ie, voxel) *x*. If we assume that *M* components are present in a voxel, the measured data *d*(*x*, *b*, *TE*) can be modeled (in the absence of noise) as

(5) $$\begin{array}{c} d\left(x,b,TE\right)=\sum_{m=1}^{M}f_{m}\left(x\right)a_{m}\left(b,TE\right) \end{array}$$

where $a_{m}\left(b,TE\right)$ is a function describing the contrast variations of the mth component, and $f_{m}\left(x\right)$ is a spatial map describing how much of the mth component is present within each image voxel.

Different data analysis methods can be distinguished from each other based on the different modeling assumptions that they make. Some of the most general unmixing approaches (including nonnegative matrix factorization,^101^, ^102^ independent component analysis,^103^ and low‐rank tensor decomposition^104^) are sometimes called blind source separation methods because they make minimal prior assumptions about $a_{m}\left(b,TE\right)$ and $f_{m}\left(x\right)$. Instead, these approaches attempt to learn all the model parameters from the data, based only on the assumptions that M is relatively small and that the components $a_{m}\left(b,TE\right)$ are simple in an appropriate way. Although such methods are straightforward to implement, can be effective at automatically decomposing the data into seemingly‐meaningful components, and have been successfully used to analyze combined diffusion‐relaxometry data,^105^, ^106^, ^107^ these approaches have some potential disadvantages. These include that they may decompose data into ambiguous constituent parts that are not biologically meaningful, and the decomposition may not be stable and/or reproducible. Such methods may also overlook short $T_{2}$ compartments due to the longer TEs required to explore the $T_{2}$‐diffusion space.

A commonly used stronger model assumes that the $a_{m}\left(b,TE\right)$ are not some arbitrary/unknown functions, but can instead be described concretely. For example, one common assumption, which we make from here onwards, is that each component is associated with independent monoexponential diffusion and relaxation decays such that

(6) $$\begin{array}{c} a_{m}\left(b,TE\right)=e^{‐bD_{m}}e^{‐TE/T_{2m}}, \end{array}$$

where $D_{m}$ is an apparent diffusion coefficient and $T_{2m}$ is a $T_{2}$‐relaxation parameter. (Note that analogous assumptions can be applied for any experiment, eg, for T1 inversion recovery we have

(7) $$\begin{array}{c} a_{m}\left(b,TE,TI\right)=e^{‐bD_{m}}e^{‐TE/T_{2m}}\left(1‐2e^{‐TI/T1}\right). \end{array}$$

We include this equation here for reference, and note that the approaches discussed in the following sections equally apply to any signal equation.) Given this assumption, we now explore three main approaches for analyzing combined diffusion‐relaxometry data:

1. Continuum modeling
2. Cumulant expansion
3. Parsimonious modeling using strong biophysical assumptions

In the next section, we discuss these three approaches in turn.

### 3.2 Current state‐of‐the‐art: Continuum modeling

Thus far, continuum modeling has been the preeminent technique for analyzing combined diffusion‐relaxometry data. Under the assumption of monoexponential decays, it is common to rewrite the data model from Equation (5) as an integral equation of the first kind ^8^ according to

(8) $$\begin{array}{c} d\left(x,b,TE\right)=\iint f\left(x,D,T_{2}\right)e^{‐bD}e^{‐TE/T_{2}}dDdT_{2} \end{array}$$

where $f(x,D,T_{2})$ is the diffusion‐relaxation correlation spectrum for a voxel in spatial location *x*. The choice to use an infinite dimensional integral equation rather than the finite discrete sum from Equation (3) is consistent with earlier methods for 1D multicomponent relaxometry,^108^, ^109^ and reflects the practical reality that we often do not have prior information about the number of components M. An alternative approach, discussed later, is to incorporate prior assumptions about tissue microstructure, enabling techniques more specific to distinct tissue types, at the cost of generality. In addition, in complicated heterogeneous tissues, the number of distinct decay parameters that are present within a voxel might be very large and effectively infinite. In the literature, this integral equation has been viewed as a special case of the Laplace transform, causing the associated inverse problem to sometimes be called an inverse Laplace transform (ILT).

Since the correlation spectrum $f(x,D,T_{2})$ is infinite dimensional and practical MRI experiments only acquire a finite number of measurements, we should not expect the ILT to have a unique solution. In MRI and NMR spectroscopy, these problems are usually resolved by choosing the “simplest” solution, ie, the unique solution that fulfills minimum‐norm least‐squares (MNLS) criteria.^110^ However, while it is straightforward to formulate the ILT within the framework of infinite dimensional Hilbert spaces and derive simple analytic expressions for the unique MNLS solution,^111^ the ILT solutions obtained in this manner are usually not very satisfying or useful. This occurs because, unlike the case for MRI and NMR spectroscopy where MNLS solutions are stable and interpretable, the presence of exponential decays in Equation (3) can make the inverse problem severely ill‐posed and highly unstable. As a result, additional assumptions must usually be imposed to get reasonable ILT solutions.

There are several constraints that have been proposed to help achieve robust and interpretable correlation spectra. A widespread approach, originating in some of the early papers on diffusion‐relaxometry,^8^ is to assume that the correlation spectrum $f(x,D,T_{2})$ should be everywhere nonnegative. This assumption can be motivated by physics and is inherited from earlier work on 1D relaxometry.^108^, ^109^ To numerically perform the inversion and estimate the spectrum, Equation (8) is discretized on grids of predefined ranges with $N_{D}$ and $N_{T_{2}}$ nonnegative components in the *D* and $T_{2}$ dimensions, respectively. The maximum and minimum grid values are chosen such that the solution is physically realistic by excluding negative fractions. The resulting matrix equation is

(9) $$\begin{array}{c} d=Af, \end{array}$$

where vectors f and d are discretized versions of $f(x,D,T_{2})$ and *d*(*x*, *b*, *TE*), respectively, and the matrix *A* is a corresponding discretization of the integral equation from Equation (8). *A* is $N_{S}$ by $N_{D}N_{T_{2}}$, *f* has length $N_{D}N_{T_{2}}$, and *d* has length $N_{S}$, the total number of MR‐encodings in the experiment. This leads to a nonnegative least‐squares inverse problem formulation that can be written as

(10) $$\begin{array}{c} \hat{f}=\underset{f\geq 0}{\text{argmin}}{\left\|Af‐d\right\|}_{2}^{2} \end{array}$$

using the standard 2‐norm. This constrained optimization problem does not have an analytic solution, but can still be solved iteratively using efficient optimization algorithms.^112^ Importantly, nonnegativity constraints have good theoretical characteristics, and existence of a unique nonnegative solution can sometimes be guaranteed in inverse problems that would otherwise have infinitely many solutions.^113^

In addition to nonnegativity, regularization constraints can also be used to stabilize the inverse problem, leading to the formulation

(11) $$\begin{array}{c} \hat{f}=\underset{f\geq 0}{\text{argmin}}{\left\|Af‐d\right\|}_{2}^{2}+\lambda R\left(f\right) \end{array}$$

where *R*(.) is a regularization penalty function that is designed to prefer solutions that have certain desirable characteristics and λ is a user‐selected regularization parameter. Common choices include:

- Regularization of the spectrum from each voxel with $R\left(f\right)={\left\|Hf\right\|}_{p}^{p}$, where H is an appropriately chosen voxelwise constraint matrix, p is a user‐selected parameter, and $norm._{p}$ denotes the standard p‐norm. Common choices of *p* include p=2 (leading to standard Tikhonov regularization and guarantees under specific conditions the existence of a unique solution to Equation (11) ^8^ and p=1 (leading to standard L1 regularization, which often results in sparser solutions). Depending on the choice of *H*, this penalty can be used to enforce the constraints that the reconstructed correlation spectrum for each voxel should have small signal energy or should have relatively smooth spectral variations. This kind of regularization is popular in combined diffusion‐relaxometry,^8^, ^58^, ^114^ and also 1D relaxometry.^108^, ^109^
- Spatial regularization that encourages the reconstructed correlation spectrum $f(x,D,T_{2})$ to be spatially smooth,^100^ suited to mapping large structures over a whole image. Unlike the previous regularization penalties (which operate voxelwise and can be used to reconstruct the spectra for each voxel separately), the use of spatial regularization couples the estimation of correlation spectra from neighboring voxels, and necessitates an imaging acquisition. This approach has been used in combined diffusion‐relaxometry,^100^ but was also used in earlier 1D multicomponent exponential modeling applications.^115^, ^116^ Estimation theoretic analysis can be used to show that spatial smoothness constraints can theoretically reduce the ill‐posedness of the ILT by orders of magnitude in both 1D^116^ and higher‐dimensional^55^ settings.
- Data‐driven regularization, where a fixed number of correlation spectra $f(x,D,T_{2})$ are assumed within the image.^117^, ^118^ This approach is related to the previously mentioned blind source‐separation methods^105^, ^106^, ^107^ and seeks a lower‐dimensional spectral representation of the image that is supported by the data, effectively regularizing the inversion by sharing information across voxels. This approach is appropriate when seeking to discover prominent microstructural features, at the expense of estimating spectra in every voxel.

We note that selection of the optimal regularization tuning parameter λ is an open research question, with many approaches and strategies suggested and tested over the course more than three decades. Notable methods include the generalized cross‐validation (GCV) method,^119^ the Butler‐Reeds‐Dawson (BRD) algorithm and the L‐curve method^112^, ^120^ and theoretical regularization parameter selection based on the desired spatial‐spectral resolution characteristics.^55^, ^121^, ^122^ A thorough review of some of these methods, along with alternative inversion methods, can be found here.^123^

In addition to nonnegativity and regularization, other potentially useful constraints include:

- Enforcing consistency between the multidimensional correlation spectrum derived from combined diffusion‐relaxometry data and the 1D diffusion and relaxation spectra derived from 1D ILTs of subsets of the data.^58^ For example, the MADCO technique—as discussed in the Acquisition section—exploits the fact that lower dimensional spectra are projections of higher dimensional spectra, for example,

(12) $$\begin{array}{c} f\left(T_{2}\right)=\int_{0}^{\infty }f\left(T_{2},D\right)dD. \end{array}$$

to enforce constraints on the multidimensional spectrum estimation.

#### 3.2.1 Mapping voxelwise spectra

Unlike modeling methods that calculate a single, or small number of, values per voxel (as discussed later), the continuum modeling approach we describe calculates the spectrum, *f*, in each voxel. The typical way to derive meaningful maps from such voxelwise spectra is known as *spectral integration*.^60^, ^100^, ^124^ In summary, spectral intergration comprises first manually identifying prominent regions of the spectrum—typically by examining a spectrum derived from the signal averaged over a large representative ROI. The proportion of each voxelwise spectra that lies within each of these prominent regions is then calculated (hence the name spectral integration), yielding scalar indices often termed apparent spectral volume fractions. The data‐driven regularization methods described above^117^, ^118^ provide an alternative approach for deriving maps.

In contrast to continuum modeling, a useful and widely used constraint in single‐contrast diffusion and relaxation experiments is to assume that the number of components M in Equation (1) is known in advance and is very small, while also assuming simple/parsimonious parametric models for the signal observed from each component. This approach has been used with combined diffusion‐relaxometry data,^26^, ^74^, ^125^ and uses modeling assumptions that are substantially more restrictive than those previously discussed. However, when the modeling assumptions are accurate, they lead to a simpler inverse problem that requires substantially lower SNR data to solve than the more general formulations described previously (calculating multidimensional spectra, particularly for single voxels, requires very high SNR). We discuss two examples of this approach in the next sections.

### 3.3 Current state‐of‐the‐art: Signal representations

As previously discussed, a major weakness of continuum modeling is that the inversion of the Fredholm integral or Laplace transform is ill‐posed.^126^ Naturally, there are representations that do not require an ILT. The most commonly used representations in diffusion MRI are diffusion tensor imaging (DTI)^127^ and diffusion kurtosis imaging (DKI)^128^ (although further techniques have been demonstrated, such as a decomposition in the basis of harmonic oscillator eigenmodes,^129^ spherical harmonics, or extended phase graph (EPG) formulation.^130^ DTI and DKI correspond to truncating the cumulant expansion,^131^ which is the Taylor expansion of logS(q) for small diffusion wave vector *q*, at the 2nd order and 4th order, respectively. Ning et al^132^ adapted the cumulant expansion approach to diffusion‐relaxation experiments, hence calculating the joint moments of relaxation rate and diffusivity. This allowed the calculation of several novel microstructural metrics, including estimating diffusion properties independently from TE without solving an ILT.

Signal representations are a potential starting point to determine the number of degrees of freedom that a future microstructural model could have. For instance, the observation of bi‐exponential decay in S(b) or a nonzero kurtosis necessarily indicates that the measurement is sensitive to non‐Gaussian diffusion.^133^ The observation of diffusion time dependence suggests that this non‐Gaussian diffusion occurs in one or more compartments.^134^ Further study of mathematical functional forms would reveal even more information about the underlying structure.

### 3.4 Current state‐of‐the‐art: Parsimonious modeling using strong biophysical assumptions

Biophysical modeling moves to a biophysical description of the signal, where we assume a fixed number of tissue compartments, essentially trying to identify the relevant degrees of freedom while discarding many others.^135^ In our notation we can write the signal as a sum over multiple tissue compartments

(13) $$\begin{array}{c} d\left(x,b,TE\right)=\sum_{i=1}^{N}f_{i}\left(x\right)a_{i}\left(b,TE\right) \end{array}$$

where we change the index from Equation (5) to reflect the fact that we assume a known number of tissue compartments, *N*. The choice of *N* and the form of each $a_{i}\left(b,TE\right)$ is biophysically motivated based on our knowledge of the tissue of interest (see Ref. [136] for a summary of typical compartments).

Multicompartment biophysical models are prevalent in diffusion MRI, particularly in the brain.^137^, ^138^ However, a significant limitation is that due to model identifiability issues, it is common to fix certain parameters to reasonable values.^139^, ^140^ Although this approach can produce high precision biomarkers, it also introduces biases.^141^, ^142^ Accounting for both the diffusion and relaxometry properties of tissue compartments during acquisition and analysis can avoid fixing parameters, as has been demonstrated in the brain^57^ and prostate.^143^ When combined with rich enough data and robust parameter estimation techniques, biophysical models have the potential to estimate highly specific physical features—such as permeabilities, compartment sizes, orientation dispersion, packing correlation length scales.

Metrics obtained from multicompartment diffusion MRI models can be directly compared to histology, since it can be reasonably assumed that the measured tissue microstructure remains reasonably similar in the ex‐vivo histology (with the caveats of membrane integrity and shrinkage due to tissue preparation,^144^ and the geometric mismatch between 3D MRI and 2D histology^145^). However, the extent to which this generalizes to diffusion‐relaxometry is a question attracting significant research interest.

### 3.5 Future promises

**FIGURE 7:**
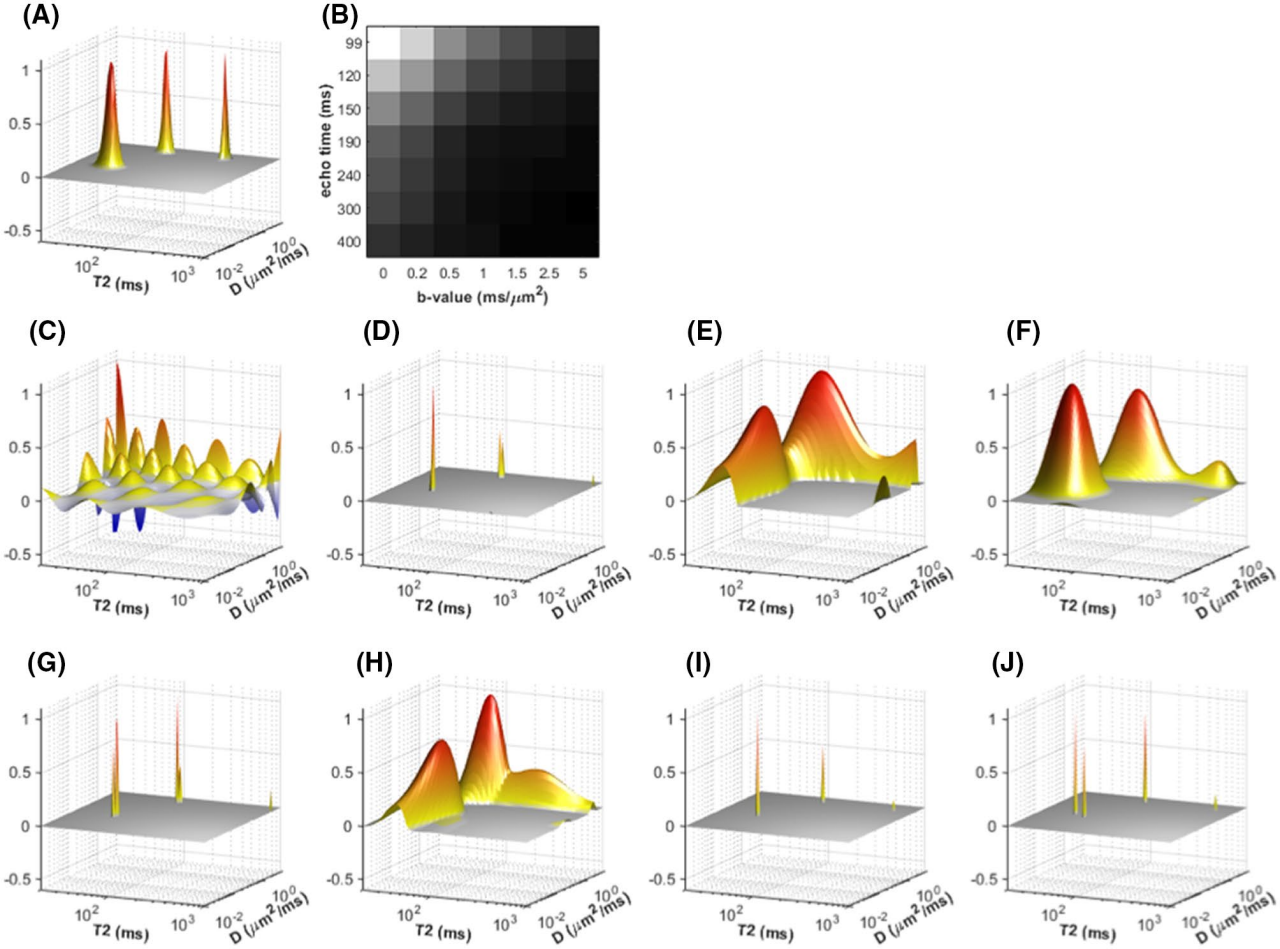
Illustration of estimating 2D diffusion‐relaxation correlation spectra using different kinds of constraints. A, Simulated ground truth spectrum from a single voxel, comprised of three spectral peaks with Gaussian lineshapes. (The simulation is identical to that described in previous work,^100^ and we omit the details). B, Simulated noisy combined diffusion‐relaxometry data from one voxel (following,^100^ the SNR for the highest‐SNR image was 200). C‐J, Reconstruction results using different kinds of constraints: C, MNLS; D, nonnegative least‐squares using Equation (4); E‐H, regularized solutions using Equation (5), including (E) voxelwise Tikhonov regularization (H is an identity matrix), (F) voxelwise Tikhonov regularization (H computes a finite‐difference approximation of second‐derivatives along the vertical and horizontal dimensions of the spectrum), (G) voxelwise L1‐norm regularization (H is an identity matrix), and (H) spatially regularized reconstruction (assuming the spatial distribution described in previous work^100^). We also show fits obtained using Equation (1) with known M and the parametric model from Equation (2) for each component: (I) M=3 and (J) M=4. Note that these spectra can look very different from one another even though they are all similarly consistent with the measured data (less than 0.4% data error in all cases). This reflects the severe ill‐posedness of the problem and the need to choose constraints carefully. As can be seen, the MNLS reconstruction completely fails to capture the true structure of the data, while the remaining constrained reconstructions have varying levels of correspondence with the ground truth spectrum. Note that it is very hard for any of these methods to estimate the lineshapes of the spectral peaks accurately (some have much sharper peaks while others have much broader peaks), and that aside from (H), the rest of the reconstructions have spectral peaks in the wrong locations and potentially incorrect spectral peak integrals. Despite these mismatches with the ground truth, many of these estimation methods have been shown to produce consistent spectral decompositions of real data that can serve as useful biomarkers for different microstructural characteristics

As can be seen, there are many different options available for the analysis of combined diffusion‐relaxometry data that have complementary strengths, with different methods being more or less powerful in different application contexts. For illustration, the effects of using different kinds of estimation constraints are shown using synthetic diffusion‐relaxometry data in Figure 7. This represents a specific toy $T_{2}$‐diffusion system where the ground truth for each microenvironment is represented by a simple Gaussian distribution. In reality, microenvironments will have more complex, and potentially overlapping, shapes. In cases where the $T_{2}$‐diffusion approach cannot disentangle the relevant microenvironments, the system could be explored with higher‐dimensional acquisition approaches detailed in this review, such as $T_{1}$‐$T_{2}$‐diffusion, incorporating diffusion time dependence, etc.

While the approaches described above can be quite powerful, it should be emphasized that this is still a developing field, and there are many opportunities to make combined diffusion‐relaxometry data analysis even better. For example, recent literature has demonstrated that it is possible to quantify the estimation uncertainty associated with combined diffusion‐relaxometry experiments using Cramer‐Rao theory,^55^, ^57^, ^146^ similar to approaches used in other quantitative MRI applications.^147^, ^148^, ^149^ While such uncertainty quantification is clearly useful for interpreting experimental results, this kind of information is also important because it can be used to design more efficient data acquisition schemes.^57^, ^146^ As further examples, it has recently been shown that a nonnegativity‐constrained ILT formulation can be combined together with statistical resampling methods to enable very high‐dimensional correlation spectrum estimation without additional regularization constraints.^150^ We also note that we assumed time‐independent diffusion with no intra‐compartmental kurtosis in Equation (6). Mean kurtosis can be estimated by adding the relevant terms to Equation (6),^128^ or different sources of kurtosis could be decoupled by combining with new approaches such as correlation tensor imaging.^151^ There are also potentially many other creative approaches that are yet to be developed. Machine learning is a promising avenue; this will likely focus on methods that learn from simulations (eg, Refs. [152, 153]) or unsupervised learning (eg, Ref. [118]), as ground truth information on tissue microstructure is not typically obtainable.

## 4 APPLICATIONS

### 4.1 Brain

#### 4.1.1 Background and motivation: Brain applications

There are a wide range of diffusion MRI brain microstructure imaging techniques, many of which show promise in the clinic.^154^ Compared to relaxometry and more conventional DTI, the main advantage offered by these techniques is a more direct link between quantitative parametric maps and the underlying tissue microstructure. For example, simple diffusion models have shown remarkable value for imaging acute ischemic stroke,^155^ but the exact microstructural changes driving the contrast in ADC and DTI maps remain unclear. Moreover, unaccounted for changes in relaxation properties of brain tissue due to pathological conditions (eg, increase of $T_{2}$ relaxation time in ischemic lesion^156^) may bias parameter estimates and lead to misleading or wrong interpretation of the ongoing pathological mechanisms. Incorporating relaxation into diffusion MRI techniques can lead to more specific measures of brain microstructure, leading to improved understanding of disease and new clinical applications.

#### 4.1.2 Current state‐of‐the‐art: Brain applications

There are a wide range of brain microstructure models, spanning signal representations and biophysical models.^138^ The most common brain microstructure model encompasses a large set of white matter models consisting of Gaussian compartments, is the so‐called “Standard model”.^135^ This describes the measured dMRI signal as the convolution of a fiber orientation distribution function (ODF), $P(\hat{n})$, and a response kernel, $K(b,\hat{g}\cdot \hat{n})$ from a straight fiber oriented along $\hat{n}$, where $\hat{g}$ is the unit gradient direction:

(14) $$\begin{array}{c} S_{\hat{g}}\left(b\right)=\int_{‐\infty }^{\infty }d\hat{n}P\left(\hat{n}\right)K\left(b,\hat{g}\cdot \hat{n}\right) \end{array}$$

while the Standard model does not fix individual parameters,^26^, ^57^, ^157^ there are numerous particular cases of this model that involve prior assumptions or bounds on the scalar parameters of the kernel $K(b,\hat{g}\cdot \hat{n})$ (diffusivities, volume fractions), inclusion of free water compartment, and assumptions relating to the functional form of the ODF, most generally represented by an expansion of spherical harmonic coefficients. Fixing compartment diffusion coefficients and the ODF shape introduces an unknown a priori bias^141^ on the remaining parameters, which is particularly problematic in regions with diffusivity changes and no change in the volume fractions of tissue compartments.^158^ On the other hand, without any priors or constraints the standard model suffers from degeneracy, that is, multiple combinations of the model parameters can provide the same indistinguishable signals.^141^ Simultaneously measuring relaxation and diffusion properties can help address this issue and relax a number of the priors and constraints. This was recently demonstrated in the brain, where an optimized 5D protocol comprising multiple TE and b‐tensor shapes was shown to enable the fit of a minimally constrained white matter model.^57^

**FIGURE 8:**
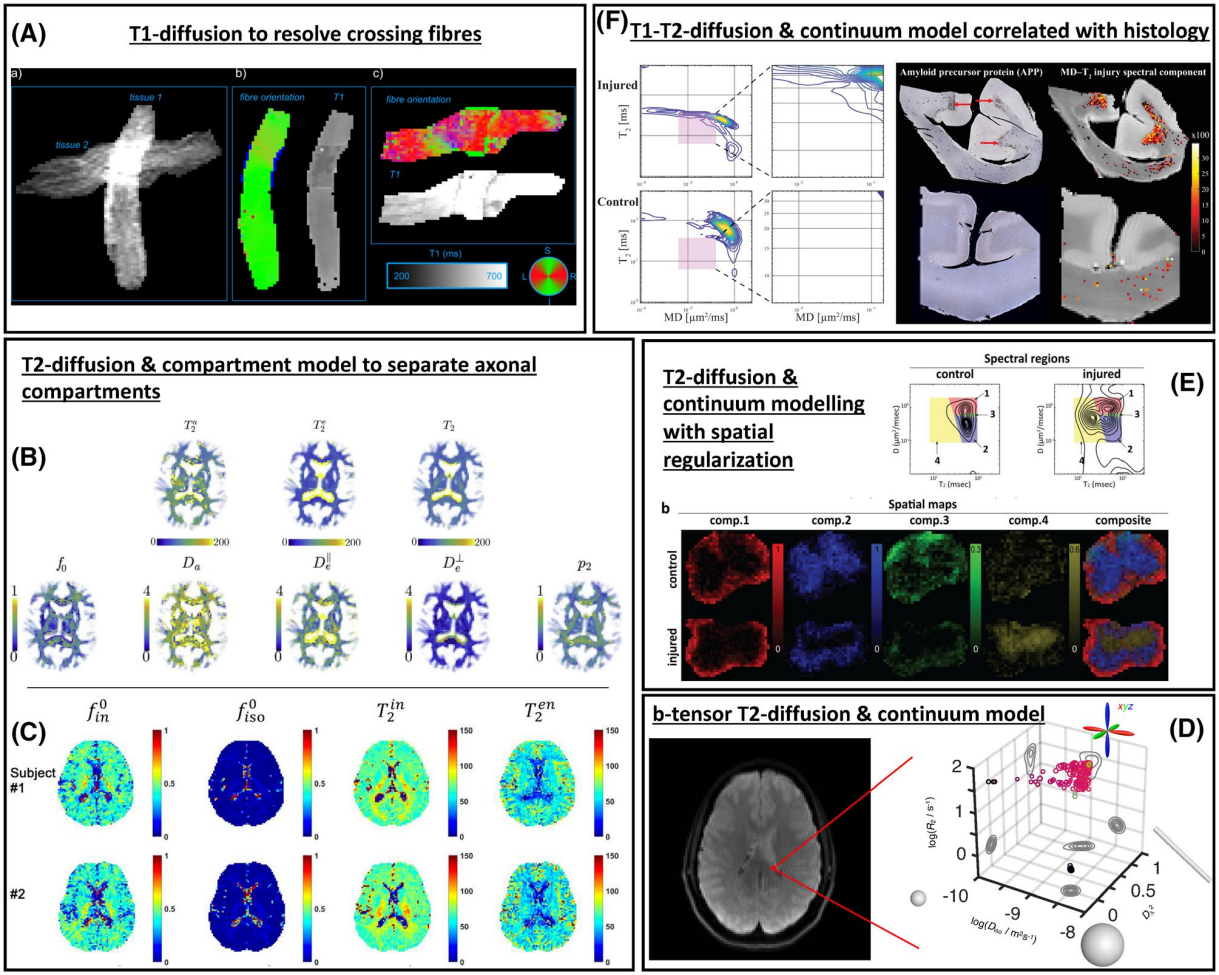
Examples of combined diffusion‐relaxometry in brain and spinal cord applications. A, $T_{1}$‐diffusion—direction encoded color map $T_{1}$ maps of tissue in a fiber crossing phantom from Ref. [125]. B, $T_{2}$‐diffusion—TE‐dependent diffusion imaging (TEdDI) maps for in vivo brain from Ref. [26]. Top row: intra‐ and extra‐axonal $T_{2}$, and mean $T_{2}$; bottom row: diffusivity parameters. C, $T_{2}$‐diffusion—Multi‐TE NODDI parameter maps for in vivo brain from Ref. [29]. Parameters from left to right are: intraneurite fraction, free water volume fraction, and intra‐ and extra‐neurite $T_{2}$ values. D, $T_{2}$‐diffusion with b‐tensor encoding—5D diffusion‐relaxation distribution for a selected voxel of in vivo brain scan from Ref. [161]. E, $T_{2}$‐diffusion—spatially averaged $T_{2}$‐D distributions and spectral volume fraction maps in control and injured spinal cord samples from Ref. [100]. F, $T_{1}$‐ $T_{2}$‐diffusion—spectral signature of microscopic lesions in the human corpus callosum (top: injured, bottom: control). Right panel shows corresponding injury biomarker images, side‐by‐side with histology.^164^ Subfigures A, C, and D are reproduced under Creative Commons licenses; subfigures B, E, and F are used with permission

Both $T_{1}$ and $T_{2}$ have been utilized to resolve inherent ambiguities in diffusion measurements. De Santis et al used combined $T_{1}$‐diffusion acquisition to resolve crossing fiber populations^31^, ^125^ (see Figure 8A). More recently, Leppert et al investigated tract specific $T_{1}$ mapping.^40^ Veraart et al used combined $T_{2}$‐diffusion to improve separation of presumed intra‐ and extra‐axonal compartments^26^ (Figure 8B), and a similar strategy was later adopted within the NODDI model framework^29^ (Figure 8C). An interesting different multidimensional approach has been recently proposed to map intra‐axonal $T_{2}^{*}$ values using the extra‐axonal water suppression provided by high diffusion‐weighting. Using a diffusion‐filtered asymmetric spin echo, Kleban et al jointly estimated diffusion and susceptibility effects in major brain white matter tracts, showing that intra‐axonal $T_{2}^{*}$ is lower in the corticospinal tract than in corpus callosum and cingulum.^159^

Moving away from biophysical model‐based approaches, more data‐driven methods to multidimensional diffusion‐relaxation analysis of the brain tissue have been recently proposed, carrying very exciting perspectives (for a more comprehensive review of the most recent methods, we refer the reader to Refs. [55, 160]). For example, de Almeida Martins et al proposed an MRI framework to quantify the microscopic heterogeneity of the living human brain by spatially resolving five‐dimensional relaxation‐diffusion distributions from combined 5D relaxation acquisition,^161^, ^162^ with experiments on a healthy volunteer demonstrating that the retrieved 5D $T_{2}$‐D distributions can resolve distinct microscopic tissue environments within the living human brain (see Figure 8D). It is worthwhile to mention here that these data‐driven multidimensional diffusion‐relaxation approaches can be extremely useful to characterize parts of the central nervous system other than the brain, such as the spinal cord^100^, ^163^ (eg, Figure 8E). Recently, in a comprehensive postmortem multidimensional MRI and histopathology combined study that included multiple human subjects ranging in axonal injury severities, Benjamini et al showed that axonal injury has a multidimensional spectral signature,^164^ which can be used to generate injury biomarker images that closely follow amyloid precursor protein (APP, axonal injury marker) histopathology, with strong correlation (see Figure 8F).

Of course, more general data‐driven multidimensional approaches come together with new challenges,^160^ for example, how to improve the robustness (in terms of accuracy and precision) of the statistical descriptors that are estimated directly from the data,^165^ or how to summarize and visualize in an effective and simple way the large amount of information that can be retrieved.^161^, ^166^ A particularly interesting model‐free application of multidimensional relaxation‐diffusion approach to the same problem previously tackled by De Santis et al using a model‐based approach has been recently proposed for the nonparametric estimation of fiber‐specific diffusion‐$T_{1}$ features.^41^ However, for spinal cord, the application of such methods to in vivo clinical settings is hampered by challenges involving low SNR and high motion corruption. Nevertheless, recently proposed strategies to reduce the number of samples^167^ may provide useful tools to address some of these limitations, opening exciting possibilities for in vivo clinical applications.

#### 4.1.3 Future promise: Brain applications

Combined diffusion‐relaxometry MRI is quickly evolving and moving into more and more clinically feasible applications.^160^ As discussed in the previous sections, this new approach comes with new challenges, but several recent works have already highlighted interesting avenues toward the resolution of some of them.^161^, ^165^, ^166^ Notwithstanding the current challenges, the possibilities offered by combined diffusion‐relaxometry for brain microstructure characterization are exciting, especially for pathological conditions. In particular, the rich data available from combined diffusion‐relaxometry lend itself to data‐driven analysis (eg, continuum modeling), which in principle can better generalize across diseases than typical brain microstructure models that make a priori assumptions about tissue structure.

In addition, new acquisition techniques have the ability to probe very short TEs,^49^ paving the way toward very interesting applications, for example, the quantification of combined diffusion‐relaxation properties of myelin, whose $T_{2}$ is very short (typically <10 ms), raising in turns new challenges for the acquisition. Furthermore, advanced MRI hardware, utilizing stronger diffusion gradients such as those available in the CONNECTOM scanner,^49^ may allow the exploration of an additional dimension, the diffusion time, that may carry unique information about microstructural features such as membrane permeability, spatial disorder and more (see further the earlier Diffusion Time Section). Promising future applications may involve the multidimensional exploration of sub‐cellular membrane structures^168^ or the combined diffusion‐relaxation of brain metabolites.^169^, ^170^

### 4.2 Prostate

#### 4.2.1 Background and motivation: Prostate applications

The multiparametric MRI (mp‐MRI) examination for diagnosis, staging, and risk stratification of prostate cancer relies on $T_{2}$‐weighted and diffusion MRI images.^171^ The synergy between modalities is abundantly clear to clinicians; moreover, decoupling signals from multiple tissue compartments promises biomarkers with greater diagnostic value.^172^ This is a monumental modeling challenge, as the prostate is heterogeneous at multiple length scales, where each voxel presents unknown mixtures of stroma (20 μm), epithelium (10 μm), vasculature (10 μm), and lumen (100 μm). The observation^173^, ^174^ of a nonmonotonic diffusion time dependence, necessarily means that at least 1 compartment falls within the short or long‐time diffusion limits. For this reason, it is incredibly difficult to estimate compartment fractions via diffusion, as this would require definition of higher order cumulants of the Taylor expansion of $\log(\exp‐bD^{n}\left(t\right))$ for each compartment. A potential avenue to resolve the model complexity of diffusion is to rely on relaxometry for compartment estimation, where separation of signal into cellular (stromal + epithelium) and luminal compartments using $T_{2}$ alone has been performed reliably in prostate tissue as early as 1987^175^ and has been reproduced on numerous occasions.^145^, ^176^, ^177^, ^178^ The practical challenge remains on how to describe the prostate signal through diffusion‐relaxometry, without biasing parameter estimates or over complicating the model.

#### 4.2.2 Current state‐of‐the‐art: Prostate applications

**FIGURE 9:**
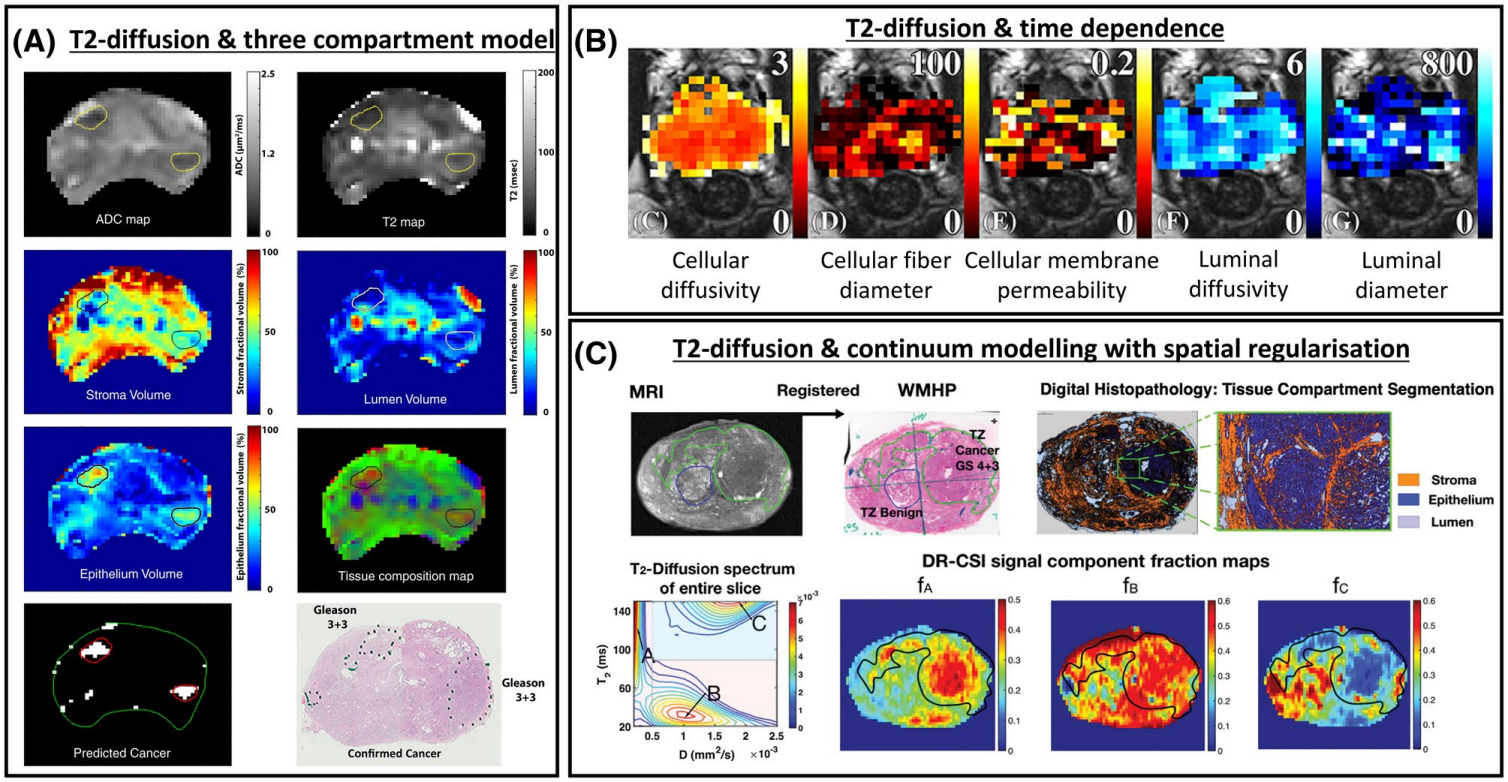
Examples of combined diffusion‐relaxometry in the prostate. A, $T_{2}$‐diffusion—MR‐derived parameter maps and predicted cancer map, and corresponding histologic maps for prostate with Gleason 3+3 cancers.^179^ B, $T_{2}$‐diffusion with varying diffusion time—parameter maps for healthy prostate from [74]. C, $T_{2}$‐diffusion—and corresponding histology for prostate with Gleason 4+3 cancer from [181]. Subfigures A and C are used with permission, and subfigure B under a Creative Commons license

The model of Chatterjee et al^179^ incorporates relaxometry and hypothesizes that prostate diffusion can be described as a sum of 3 Gaussian compartments: epithelium, stroma, and lumen (see Figure 9A). The model of Lemberskiy et al^74^ circumvents the issue of higher order cumulants, by modeling DWI at low‐b (where diffusion remains sufficiently Gaussian) and performing the assumed compartmental decomposition of the diffusion tensor entirely through $T_{2}$, thereby preserving the full diffusion tensor for each compartment (see Figure 9B). This approach allowed for the functional validation of diffusion time dependence within putative cellular (epithelium and stroma), which was found to agree with random permeable barriers, and luminal compartments were found to agree with the short‐time limit. Though high b‐values were not considered, exchange/permeability could be measured for the cellular compartment via a random permeable barrier model.^75^, ^180^ This approach did not account for vascularity, which while small, may be a critical microstructural biomarker. VERDICT does account for vascularity,^137^ it originally did not include relaxometry but was recently expanded to account for $T_{1}$ and $T_{2}$ dependence.^143^ Zhang et al recently applied continuum modeling with spatial regularization to quantify epithelium, stroma, and lumen compartments, and showed that these correlated well with results from histopathlogy^181^ (see Figure 9C).

#### 4.2.3 Future promise: Prostate applications

The complexity of individual compartments and the potential for diffusion time‐dependence present a challenge to modeling prostate microstructure. Unlike the mapping of axonal diameter in the brain,^72^ MR gradient systems are sufficiently strong to measure various microstructural features. With the hardware challenge resolved, the primary challenge is to determine the optimal combined diffusion‐relaxometry acquisition and its subsequent interpretation through appropriate biophysical modeling.

### 4.3 Placenta

#### 4.3.1 Background and motivation: Placenta applications

Placental MRI is emerging as a sensitive tool which can supplement antenatal ultrasound in the clinic. There are a number of MR contrasts which show promise as markers of placenta‐related pregnancy complications. $T_{2}^{*}$ relaxometry—which relates to oxygenation levels—reflects placental dysfunction in fetal growth restriction (FGR)^182^, ^183^ and pre‐eclampsia.^184^ Diffusion MRI is also sensitive to these changes, with ADC^185^ and IVIM‐derived perfusion fraction^186^, ^187^ decreased in FGR cases.

The placenta is unique in that it contains two distinct and nonmixing circulations: fetal and maternal blood. Fetal blood circulates in vasculature within convoluted villous tree structures. On the other hand, maternal blood, uniquely, does not flow within vasculature, but resides in intervillous space surrounding fetal villous trees. This one‐of‐a‐kind structure is a leading motivation behind applying combined diffusion‐relaxometry in the placenta. Melbourne et al make the following initial speculations about placental tissue environments^188^: fetal and maternal blood have similar relaxation times, and hence their relative fractions cannot be determined with relaxometry; However, they can likely be differentiated by diffusivity properties, since fetal blood perfusing in vasculature travels much faster than maternal blood. Moreover, maternal blood cannot be separated from water in tissue by diffusivity, but can be separated by relaxation time. It follows that diffusion‐relaxometry is necessary to adequately disentangle these respective compartments.

#### 4.3.2 Current state‐of‐the‐art: Placenta applications

**FIGURE 10:**
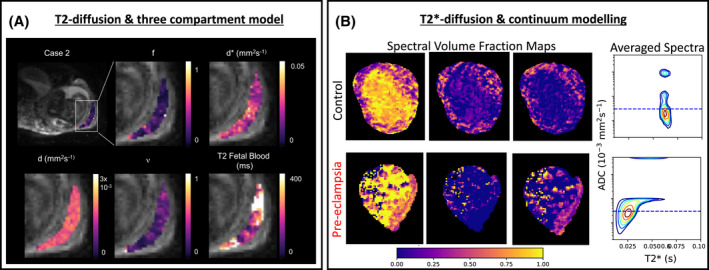
Examples of combined diffusion‐relaxometry in the placenta. A, $T_{2}$‐diffusion—parameter maps from DECIDE model fit on healthy placenta.^188^ B, $T_{2}^{*}$‐diffusion—spatially averaged $T_{2}^{*}$‐D spectra and spectral volume fraction maps in healthy and pre‐eclamptic placentas.^189^ Both subfigures are used under a Creative Commons license

The first example of combined diffusion‐relaxometry in the placenta was presented by Melbourne et al^188^ (see Figure 10A). The DECIDE model contains three putative tissue compartments: fetal blood (characterized by high diffusivity and long $T_{2}$), maternal blood (low diffusivity, long $T_{2}$), and tissue (low diffusivity, short $T_{2}$). This enables estimation of fetal and maternal blood $T_{2}$ values, and the maternal to fetal blood volume ratio. These are potential biomarkers of placental dysfunction that are unavailable through single contrast measurements.

Slator et al^189^ simultaneously probed $T_{2}^{*}$ and diffusivity in the placenta (see Figure 10B). The $T_{2}^{*}$‐ADC spectrum was calculated with an ILT, and showed multiple separated peaks potentially reflecting multiple tissue microenvironments. Encouragingly, although the sample size is small, $T_{2}^{*}$‐ADC spectra clearly separated normal and dysfunction placentas, demonstrating the potential of this approach to quantify and predict pregnancy complications.

#### 4.3.3 Future promise: Placenta applications

A promising avenue for future work is to account for anisotropy in analysis techniques. It has been shown that models incorporating anisotropy—in both perfusion and diffusion regimes—explain the placental dMRI signal well ^190^. Incorporating this into diffusion‐relaxometry analysis techniques has the potential to increase sensitivity to pregnancy complications since, for example, fetal vascular tree morphology is significantly altered in fetal growth restriction ^191^. The ultimate aim is to develop targeted imaging tools that can supplement antenatal ultrasound in the clinic. Such a tool should predict and precisely diagnose pregnancy complications, and hence be beneficial for potential future therapies. The extent to which such a tool is sensitive to the fetal to maternal blood ratio, oxygenation levels, and placental microstructure is an open question.

## 5 CONCLUSIONS

All diffusion measurements are influenced to some extent by relaxometry properties. Combined diffusion‐relaxometry measurements can leverage this intrinsic link to reveal information on tissue composition and microstructure that is inaccessible through typical separate diffusion and relaxometry experiments. Despite the challenges, such as long scanning times and multidimensional datasets, continually improving acquisition and analysis techniques can unlock the full potential of combined diffusion‐relaxometry MRI, enabling the next generation of microstructure imaging techniques.

## CONFLICT OF INTEREST

No conflicts of interest.
